# Dengue virus exploits autophagy vesicles and secretory pathways to promote transmission by human dendritic cells

**DOI:** 10.3389/fimmu.2024.1260439

**Published:** 2024-05-28

**Authors:** Alexandra P. M. Cloherty, Anusca G. Rader, Kharishma S. Patel, Tracy-Jane T. H. D. Eisden, Sterre van Piggelen, Renée R. C. E. Schreurs, Carla M. S. Ribeiro

**Affiliations:** ^1^ Department of Experimental Immunology, Amsterdam UMC, University of Amsterdam, Amsterdam, Netherlands; ^2^ Amsterdam Institute for Immunology and Infectious Diseases, Amsterdam, Netherlands; ^3^ Amsterdam Gastroenterology Endocrinology Metabolism, Amsterdam, Netherlands; ^4^ Department of Medical Oncology, Cancer Center Amsterdam, Amsterdam UMC, Vrije Universiteit Amsterdam, Amsterdam, Netherlands

**Keywords:** dengue virus, autophagy, extracellular vesicles, dendritic cells, secretory autophagy, viral transmission, viral evasion, host-directed antivirals

## Abstract

Dengue virus (DENV), transmitted by infected mosquitoes, is a major public health concern, with approximately half the world’s population at risk for infection. Recent decades have increasing incidence of dengue-associated disease alongside growing frequency of outbreaks. Although promising progress has been made in anti-DENV immunizations, post-infection treatment remains limited to non-specific supportive treatments. Development of antiviral therapeutics is thus required to limit DENV dissemination in humans and to help control the severity of outbreaks. Dendritic cells (DCs) are amongst the first cells to encounter DENV upon injection into the human skin mucosa, and thereafter promote systemic viral dissemination to additional human target cells. Autophagy is a vesicle trafficking pathway involving the formation of cytosolic autophagosomes, and recent reports have highlighted the extensive manipulation of autophagy by flaviviruses, including DENV, for viral replication. However, the temporal profiling and function of autophagy activity in DENV infection and transmission by human primary DCs remains poorly understood. Herein, we demonstrate that mechanisms of autophagosome formation and extracellular vesicle (EV) release have a pro-viral role in DC-mediated DENV transmission. We show that DENV exploits early-stage canonical autophagy to establish infection in primary human DCs. DENV replication enhanced autophagosome formation in primary human DCs, and intrinsically-heightened autophagosome biogenesis correlated with relatively higher rates of DC susceptibility to DENV. Furthermore, our data suggest that viral replication intermediates co-localize with autophagosomes, while productive DENV infection introduces a block at the late degradative stages of autophagy in infected DCs but not in uninfected bystander cells. Notably, we identify for the first time that approximately one-fourth of DC-derived CD9/CD81/CD63+ EVs co-express canonical autophagy marker LC3, and demonstrate that DC-derived EV populations are an alternative, cell-free mechanism by which DCs promote DENV transmission to additional target sites. Taken together, our study highlights intersections between autophagy and secretory pathways during viral infection, and puts forward autophagosome accumulation and viral RNA-laden EVs as host determinants of DC-mediated DENV infection in humans. Host-directed therapeutics targeting autophagy and exocytosis pathways thus have potential to enhance DC-driven resistance to DENV acquisition and thereby limit viral dissemination by initial human target cells following mosquito-to-human transmission of DENV.

## 1 Introduction

Dengue virus (DENV) is currently classified by the World Health Organization as a major public health concern, with approximately half of the world’s population at risk for infection (1). Over the past fifty years, there has been an unprecedented rise in the incidence of dengue-associated disease, and outbreaks have been of increasing frequency and magnitude (1, 2). The virus is now considered endemic in over 100 countries, with the Americas, South-East Asia, and the Western Pacific being the most seriously affected (1, 2). During the first months of 2024, several countries and territories of Latin America have reported an unprecedented surge in dengue cases. Furthermore, reports of dengue virus infection and autochthonous transmission are rapidly becoming an increasing concern in countries in mainland Europe, presumably due to the changing climate and thereby altered territory of dengue virus vectors (3, 4). As of 2022, the World Health Organization estimates that approximately 100-400 million infections occur per year, although over 80% of those are mild and asymptomatic (1). About 20% of the remaining cases encompass an acute flu-like illness that can develop into so-called ‘severe dengue’, a potentially lethal complication that requires careful management by medical professionals (1). Although the precise mechanisms underlying severe dengue pathogenesis are as yet not fully understood and likely multifactorial, the excessive release of pro-inflammatory cytokines by immune cells known as ‘cytokine storm’ during DENV disease progression as well as the antibody-dependent enhancement in secondary DENV infection are considered major culprits (5–7).

DENV is primarily transmitted to humans through the bite of infected female *Aedes aegypti* mosquitoes, and to a lesser extent *Aedes albopictus* mosquitos – both of which are well-adapted to urban environments and are daytime feeders (1, 8, 9). Upon the bite of an infected mosquito, dendritic cells (DCs) residing at the area of transmission are amongst the first human cells to encounter the injected virus and promote systemic viral infection (10–14). Thereafter, DENV further spreads to infect macrophages, endothelial cells, and monocytes in blood vessels and eventually peripheral organs.

Upon encountering target cells, the virus attaches to host molecules displayed on the cell surface. In the case of DCs, the viral envelope protein (E) attaches to the C-type lectin receptor DC-SIGN (15, 16). Alongside the E protein, the four distinct but related DENV serotypes also share the membrane (M) and capsid (C) structural antigens, the latter of which binds to the positive-sense single-stranded RNA (+ssRNA) viral genome (2, 16, 17). After receptor-mediated endocytosis, DENV is internalized into intracellular vesicles. This is followed by intravesicular acidification, which triggers structural changes in the viral M protein, permitting fusion of the viral envelope with the host endosome membrane and thereby release of the viral genome into the host cytosol. The +ssRNA viral genome can then directly function as mRNA template in order to generate early viral proteins, including the nonstructural protein NS3. Several early viral proteins then orchestrate the virus replication cycle, including replication of the +ssRNA viral genome via a double-stranded viral RNA (dsRNA) intermediate, and in doing so extensively re-organize host intracellular membranes (18–20).

Autophagy is primarily known as a tributary to the lysosomal degradative pathway and is characterized by the breakdown of intracellular cargo including viruses and viral components via its enclosure within specialized vesicles known as autophagosomes. Autophagosomes are recognizable by a characteristic double-membrane that is studded on both leaflets with the host molecule microtubule associated protein 1 light chain 3 (LC3)-II (21, 22). Autophagosome formation is controlled by a family of core ATG proteins, including transmembrane proteins like ATG9 that orchestrate the movement of lipids to form nascent autophagosomes (23), and the ATG5-ATG12-ATG16L1 complex which conjugates LC3 to phosphatidylethanolamine within growing autophagosome membranes thereby driving autophagosome elongation and closure (24). Subsequent fusion of autophagosomes with lysosomes, resulting in intravesicular acidification and an influx of host proteases, leads to the degradation of the enveloped contents, including both the cytoplasmic cargo and intraluminal autophagy receptors such as ubiquitin binding protein p62 (also known as sequestosome 1) (25–27). Recently, an alternative fate for autophagosomes has been described in primary human monocytes, intestinal epithelial cells, and human cell lines such as the THP-1 line (25, 28–31). This so-called secretory autophagy pathway results in export of vesicle-sequestered cytoplasmic cargo, such as IL-1β, FABP4 and lysozyme (28, 30–32), to the extracellular milieu. Recent studies highlight that core ATG proteins, such as ATG5, ATG16L1 and LC3, are pivotal in these processes of secretory autophagy and that dedicated TRIM and SNARE proteins facilitate autophagy-dependent unconventional secretion of immune modulators into the extracellular matrix (28, 29, 32–37).

During the replication cycles of DENV and other flaviviruses, host autophagy is extensively interfered with in order to promote remodeling of host cell lipids to provide membranous structures suitable for viral replication (18, 20, 38, 39). Flavivirus-induced lipophagy, or the selective autophagic degradation of lipid droplets, is proviral, likely because it provides energy necessary for viral replication (18, 40). Following DENV entry into the human cell via the endosomal route, which relies on acidifying vesicles for viral fusion with host membranes (16), it is possible that at early stages of the viral life cycle an increased number of acidifying autophagy vesicles could boost viral fusion with host membranes. This could partially underlie observed differences in DENV internalization across different human cell lines (16). On the other hand, host autophagy receptor ubiquitin binding protein p62 has been shown to target DENV viral components for autophagy-mediated lysosomal degradation in 293T and Huh7 human cell lines (41, 42). At late stages of viral replication, viral glycoproteins and host membranes encapsulate newly made viral proteins and viral RNA, forming immature DENV particles (18, 43–45). As they are transported via the trans-Golgi network towards the host cell membrane, DENV particles mature and are eventually exocytosed from host cells. Recent studies have suggested that host extracellular vesicle (EV) secretion pathways, including autophagy-associated mechanisms, may also be hijacked to support DENV replication and egress in mosquito and human cell lines (9, 46–48).

DCs are well-established as an important early target cell of dengue virus which secrete cytokines, chemokines, and interferons upon contact with DENV in order to mount an inflammatory response (10–14, 49). However, the impact of autophagy pathways and secretory vesicles on DENV replication and dissemination by human DCs remains elusive. Recently, research from our lab has highlighted the potential of targeting autophagy as a host-directed therapeutic strategy to intervene in replication and transmission of epidemic and pandemic viruses, namely HIV-1 and SARS-CoV-2 (26, 50). IV-1 actively blocks autophagy post-entry in DCs (27, 51), and autophagy-enhancing drugs decrease HIV-1 transmission by DCs thus underlining the antiviral functioning of autophagy (26). We have also recently shown that intrinsically enhanced autophagy correlates with superior antiviral T cell immunity during chronic HIV-1 infection (52). In the case of SARS-CoV-2, our research indicates that acidifying autophagy vesicles are usurped to promote viral entry (50), and additional reports indicate that the virus institutes a late block in autophagy flux to avoid autophagic degradation (53). For flaviviruses such as DENV, triggering of autophagy pathways have been variably associated with either a pro- or anti-viral role in different cellular models (18, 20, 41, 42).

Herein, we showcase the impact of autophagy dynamics on DENV infection of human DCs. DENV utilizes early stages of ULK1-dependent autophagy and autophagosome formation, whilst blocking the late degradative stages of autophagy, during viral replication in primary human DCs. Furthermore, our study highlights the intersection between autophagy-associated vesicles and exocytosis pathways, and we demonstrate a role for EV populations in facilitating DC-mediated DENV transmission to adjacent cells. Altogether, our data underscores the pro-viral roles of host autophagy pathways and extracellular vesicles in DENV-infected human DCs.

## 2 Results

### 2.1 Dengue virus exploits early stages of autophagy to establish infection in human dendritic cells

**Figure 1:**
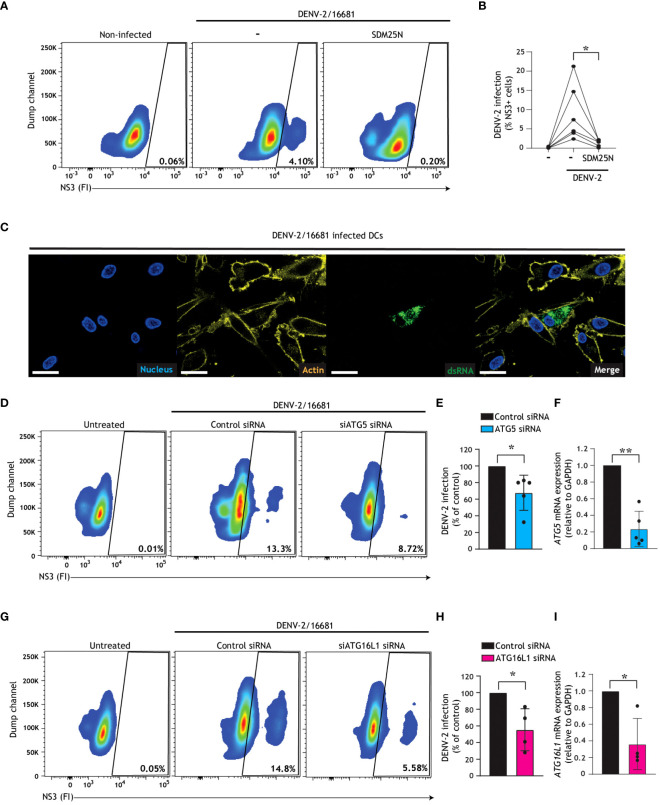
Dengue virus misuses host autophagy machinery to establish infection in human DCs. **(A-C)** Primary human DCs were infected with DENV-2/16681, with or without treatment of SDM25N replication inhibitor (10 µM) for 48 h. Representative flow cytometry plots **(A)** and quantification **(B)** of viral infection, determined by intracellular NS3 staining. Closed circles represent the mean of *n*=6 donors measured in duplicate; *P < 0.05, student’s *t*-test. **(C)** Confocal microscopy analyses of primary human DCs infected with DENV-2/16681 determined by staining for viral dsRNA (green), F-actin (yellow) and Nuclei (blue). Scale bar = 20 micron, Representative of *n* = 3. **(D, E, G, H)** Viral infection of DCs upon transfection with siATG5 **(D, E)** or siATG16L1 **(G, H)**, or non-targeting control siRNA as control **(D, E, G, H)**, followed by exposure to DENV-2/16681 for 48 h. Representative flow cytometry plots **(D, G)** and quantification of DENV-2 infection **(E, H),** determined by intracellular NS3 staining. **(F, I)** ATG5 **(F)** or ATG16L1 **(I)** silencing efficiency was determined by real-time PCR. mRNA expression was normalised to GAPDH and set at 1 in cells transfected with control siRNA. **(E, F, H, I)** Closed circles represent the mean of *n*=4-5 donors measured in duplicate; *P < 0.05, **P < 0.01, one-sample *t*-test.

Human DCs are one of the first immune cells to encounter DENV upon a mosquito bite, and due to their migratory capacity and instruction of T cell responses they have been implicated in the facilitation of DENV systemic infection and dengue associated inflammation (11–14). We and others have shown that autophagosomes and autophagy pathways play key defensive or pro-viral roles during viral replication depending on the virus in question (26, 38, 41, 46, 50), however the role of autophagy pathways during DENV replication in primary human DCs remains unexplored. First, we confirmed that primary human DCs were infected with DENV-2/16681, as indicated by the presence of the early viral non-structural protein NS3 (**Figures 1A, B**) and viral dsRNA (**Figure 1C**), the latter of which forms as an intermediate during replication of the +ssRNA viral genome. Reduction of NS3 upon treatment with SDM25N, a flavivirus replication inhibitor, corroborates that human DCs are productively infected with DENV-2 (**Figures 1A, B**). Next, in order to investigate the role of early stages of autophagy in the dengue virus replication cycle within primary human DCs, we employed RNA interference (RNAi) technology. Notably, the knock-down of either ATG5 (**Figures 1D–F**, **Supplementary Figure 1A**) or ATG16L1 (**Figures 1G-I**, **Supplementary Figure 1B**), essential autophagy molecules for autophagosome formation (54), led to a reduction in dengue virus infection of DCs. Similarly, a decreased DENV infection was observed after silencing of Atg13 or FIP200 (**Supplementary Figures 1C, D**), suggesting that ULK-1-dependent autophagy (55, 56) supports infection of DCs. Together, these results indicate that canonical autophagy mechanisms essential for the early stages of autophagosome formation promote establishment of DENV-2 infection in human DCs.

### 2.2 Dengue virus replication in DCs triggers autophagosome formation and colocalization with LC3+ autophagy vesicles

**Figure 2:**
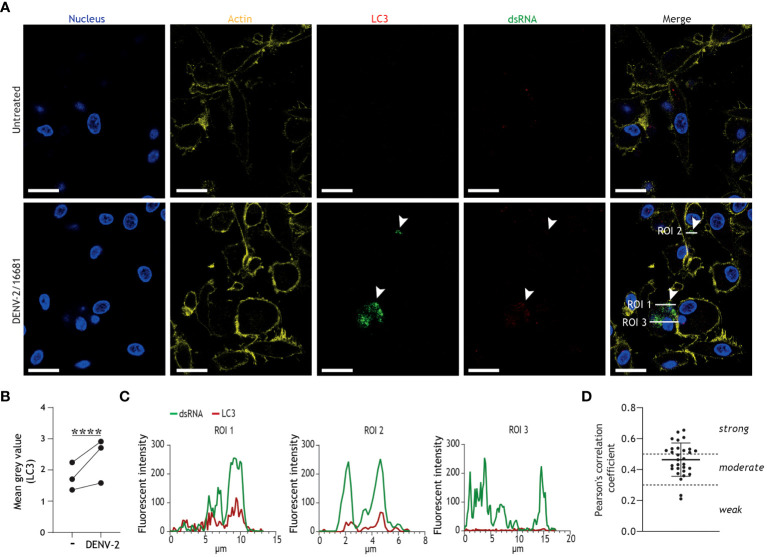
Productive infection of DCs results in increased autophagosome formation and colocalization of DENV replication intermediates with LC3+ autophagy vesicles. **(A-D)** Confocal microscopy analyses of primary human DCs infected with DENV-2/16681, or mock infected, for 48h, determined by staining for nuclei (blue), F-actin (yellow) LC3 (red), and viral dsRNA (green). Scale bar = 20 micron. **(A)** Representative confocal images. **(B)** Analysis of LC3 fluorescence; closed circles represent mean grey value across 10 fields of view for *n* = 3 donors. **(C)** Histograms of LC3 and viral dsRNA fluorescence intensities across three regions of interest (ROI) indicated in **(A)**. **(D)** Pearson’s correlation coefficient for colocalization analyses on 10 ROIs per donor between viral dsRNA and LC3, *n* = 3 donors. **(A-C)** Data are representative of *n* = 3 donors.

Next, we employed confocal imaging techniques to visualize the formation of autophagy vesicles in infected DCs, and to examine the intersection between autophagy vesicles and dengue virus replication. We observed an increase of LC3+ puncta, indicative of autophagosome formation, in DENV-infected DCs as compared to untreated controls (**Figures 2A, B**). Further in-depth analysis across three different regions of interest (ROIs; **Figures 2A, C**) demonstrated partial colocalization between dsRNA, representative of replicating dengue virus, and LC3+ vesicles (**Figure 2C**). Quantification by Pearson’s correlation coefficient analyses demonstrated moderate to strong colocalization between viral RNA and autophagosomes (**Figure 2D**). Together, these results indicate that productive DENV-2/16681 infection activates autophagosome formation, and that dengue virus replication intermediates are associated with autophagosomes in primary human DCs.

### 2.3 Impairment of autophagy flux upon productive dengue virus infection of DCs

**Figure 3:**
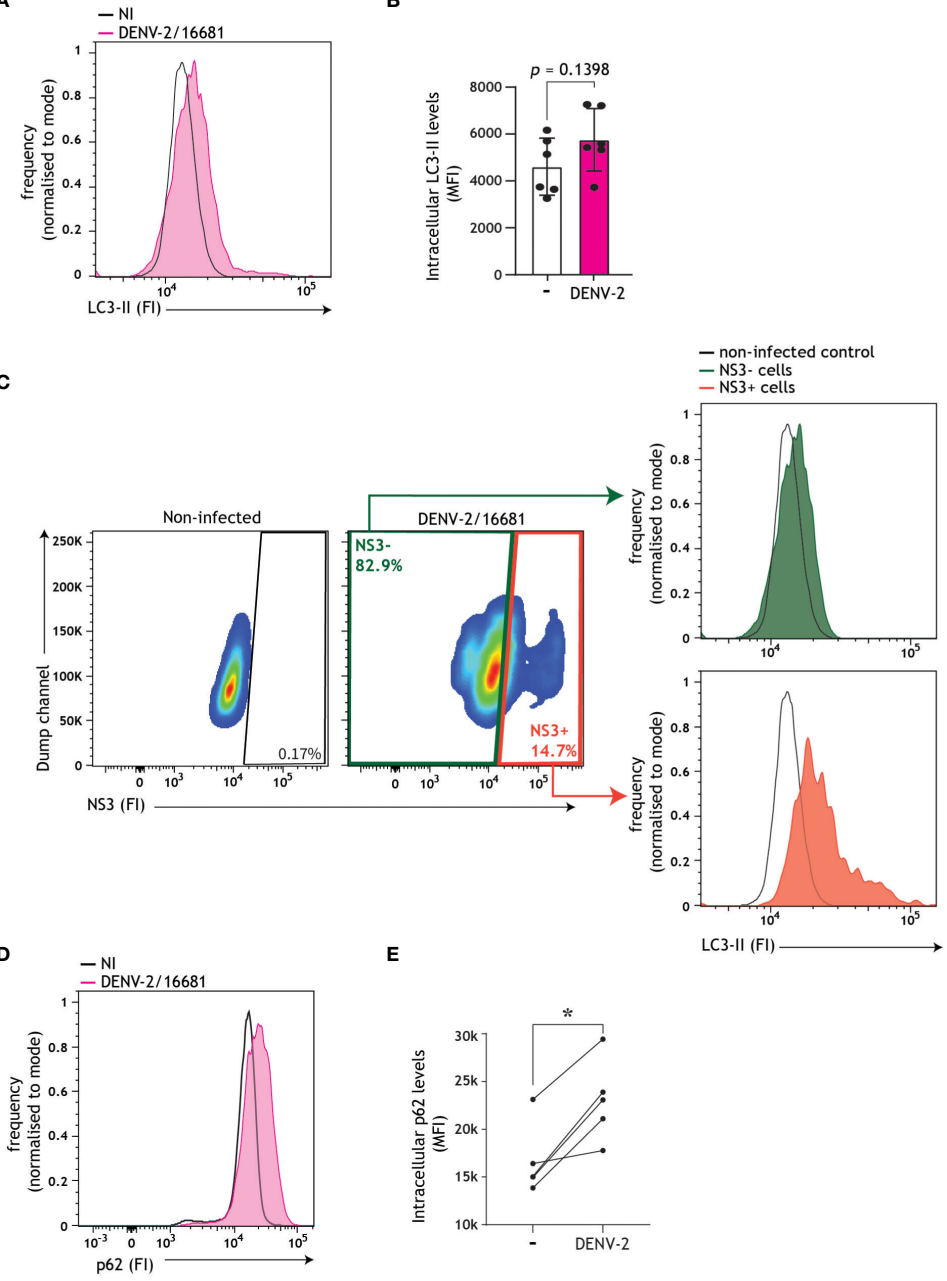
Autophagy flux impairment in DENV-infected DCs. **(A-C)** Autophagosome levels in primary human DCs infected with DENV-2/16681 for 48 h. Representative flow cytometry plots **(A)** and quantification **(B)**, determined by intracellular LC3 staining. Closed circles represent the mean of *n* = 6 independent donors measured in duplicate. **(C)** Autophagosomes levels in dengue-infected (NS3+) versus bystander (NS3-) DCs, using a concurrent intracellular NS3 and LC3 staining. Representative flow cytometry plots for *n*=2 donors measured in duplicate. **(D, E)** Intracellular p62 levels in primary human DCs infected with DENV-2/16681 for 48h. Representative flow cytometry plots **(D)** and quantification **(E)**, determined by intracellular p62 staining. Closed circles represent the mean of *n*=5 donors measured in duplicate; *P < 0.05, student’s *t*-test.

Having shown that replicating dengue virus intersects with autophagy vesicles, and both boosts and exploits autophagosome formation in order to establish infection in DCs, we next set out to investigate whether these autophagosomes follow the canonical autophagy-mediated degradative pathway, by monitoring autophagy flux in accordance with standardized best practice guidelines (57, 58). Autophagy flux, i.e. the rate of autophagosomes-lysosome fusion through the degradative process, can be monitored by detection of autophagosome-associated molecules such as LC3-II or p62. LC3-II is associated with both the inner and outer leaflets of autophagosomes, and consequently luminal LC3-II is degraded together with cargo (38). Accumulation of intracellular LC3-II levels in infected cells in the presence of autophagosome-lysosome fusion inhibitor bafilomycin A1 is indicative of enhanced autophagy flux (26, 27, 52, 58). In DCs, DENV infection did not result in induction of autophagy flux, as evident from the lack of LC3-II accumulation in DENV/bafilomycin A1-treated cells when compared to bafilomycin A1-treated cells using flow cytometric and immunoblotting analyses (**Supplementary Figures 2A, B**). In concordance with confocal imaging demonstrating increased LC3 puncta in DENV-2/16681-infected versus non-infected cells (**Figures 2A, B**), a trend towards intracellular LC3 accumulation in DENV-infected DCs in comparison to non-infected DCs was observed by flow cytometry analysis (**Figures 3A, B**). Furthermore, in-depth analyses revealed a more prominent accumulation of LC3-II in NS3+ positive cells, and to a lesser extent in NS3- bystander cells, versus non-infected controls (**Figure 3C**). Thus, the increased steady-state LC3-II levels, LC3+ puncta formation in infected cells in combination with a lack of LC3-II turnover using bafilomycin treatment, suggest that the generated autophagosomes in DENV-infected DCs do not follow the canonical late degradative stages of autophagy. To further investigate the impact of DENV infection on the late degradative stages of autophagy, we examined an additional marker of autophagy flux, namely p62. p62 is an autophagy receptor solely associated with the autophagosome inner leaflet, which links ubiquitinated cargo to the LC3-II coating autophagosome membranes, thereby selectively targeting cargo for degradation (59). Consequently, p62 is degraded together with the cargo and thereby a decrease in intracellular p62 levels is suggestive of a functional autophagy flux (58, 59). Here, we demonstrate that dengue virus infection of primary human cells results in a dramatic accumulation of intracellular p62 (**Figures 3D, E**), which is abrogated upon treatment with flavivirus replication inhibitor SDM25N (**Supplementary Figure 2C**). These results support DENV replication-dependent block in autophagy flux, as evident by accumulation of p62 as well as LC3-II in productively infected cells, leading to buildup of autophagosomes in DENV-infected DCs.

### 2.4 Intrinsically higher numbers of autophagosomes in genotyped human DCs correlates with increased DENV infection

**Figure 4:**
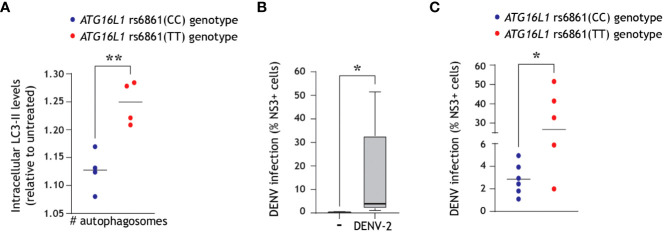
Intrinsically higher number of autophagosomes correlates with increased dengue virus infection of primary human DCs. **(A)** *ATG16L1* rs6861 (CC, blue, versus TT, red) genotyped blood-derived CD11c+ human DCs (52) were treated with bafilomycin A1 (50 nM) for 4 h prior to quantification of intracellular autophagosomes by multiparameter flow cytometry with intracellular LC3 staining combined with immune cell surface markers. DCs were defined as CD3-CD19-CD14-CD16-CD56-CD11c+ cells. Closed circles indicate quantification of intracellular LC3-II levels in *n=*4 donors for each *ATG16L1* genotype, shown as LC3-II accumulation in bafilomycin-treated cells relative to untreated cells of the same genotype. **(B, C)** Primary human DCs were infected with DENV-2/16681 for 48 h, and viral infection was quantified by flow cytometric analysis of intracellular NS3 staining. **(B)** Data represent mean % NS3+ cells across *n*=11 donors measured in duplicate. **(C)** Differentiation of viral infection levels in genotyped DCs: *ATG16L1* rs6861(CC) donors (blue-coloured circles) versus *ATG16L1* rs6861(TT) donors (red coloured circles); closed circles represent the mean of *n* = 11 donors measured in duplicate. *P < 0.05, **P < 0.01, student’s *t*-test.

We have recently identified a naturally occurring single nucleotide polymorphism (SNP) in the core autophagy gene *ATG16L1* essential for autophagosome biogenesis, namely the rs6861(TT) genotype, which is phenotypically associated with intrinsically enhanced autophagy and increased generation of autophagosomes as compared to the higher-frequency rs6861(CC) genetic variant (52). First, we confirmed herein that *ATG16L1* rs6861(TT) genotyped CD11c+ DCs display a higher number of autophagosomes as compared to rs6861(CC) genotype, by multiparameter flow cytometer in combination with LC3-II staining (**Figure 4A**). Next, we exposed DCs derived from 11 different donors to DENV-2/16681. In line with previous reports (13, 49, 60), we observed a considerable spread in the rate of infection across the different donors (**Figure 4B**). Further differentiation of these donors based on their genotype revealed that the dengue virus infection levels in genotyped DCs were relatively higher in *ATG16L1* rs6861(TT) donors as compared to *ATG16L1* rs6861(CC) (**Figure 4C**). Although we cannot infer causality between autophagy activity and DENV infection levels in these genotyped donors, these findings further suggest that an intrinsically higher abundance of autophagosomes, in combination with the observed DENV-induced autophagy flux blockade in primary DCs (**Figures 3B, C, E**), is associated with higher susceptibility for dengue virus infection in human DCs.

### 2.5 Pro-viral effect of DC-derived conditioned medium on DENV transmission

**Figure 5:**
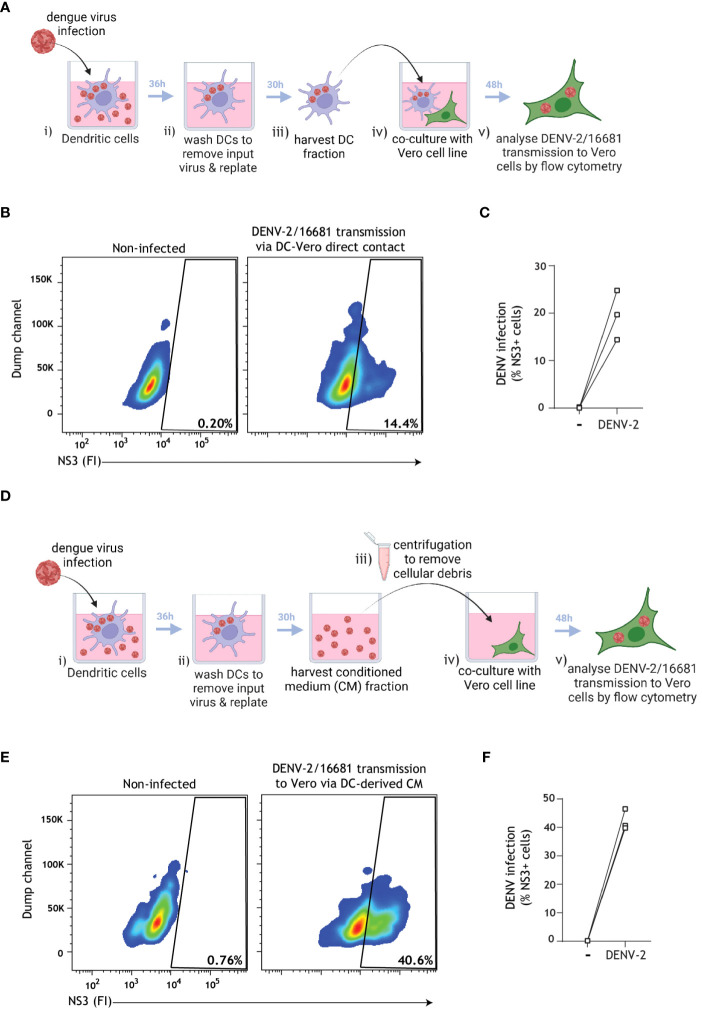
Cell-depleted supernatant derived from infected DCs mediate DENV transmission to target cells. **(A)** Graphical representation of the experimental strategy utilized to determine dengue virus transmission via cell-cell close contact in **(B, C)**. **(B, C)** Primary human DCs were infected with DENV-2/16681, or mock infected, for 36 h. DCs were then extensively washed to remove input virus, replated for 30 h, then rigorously washed and co-cultured with the susceptible Vero cell line for 48 h. DENV-2/16681 transmission by DCs to Vero was assessed in DC-Vero co-culture, determined by intracellular NS3 staining by flow cytometer. DC marker CD11c was used to exclude DCs from analysis. Data are representative flow cytometry plots **(B)** and quantification **(C)** of *n*=3 donors measured in duplicate, represented by open squares. **(D)** Graphical representation of the experimental strategy utilized to determine dengue virus transmission via DC-derived cell-free supernatant in **(E, F)**. **(E, F)** DCs were infected with DENV-2/16681, or mock infected, for 36 h. DCs were then extensively washed to remove input virus, and replated for 30 h. CM was then harvested, centrifuged to discard remaining DCs and cellular debris, and DC-free CM was transferred to Vero cell line culture. DENV-2/16681 transmission by DC-derived CM was assessed in DC-free Vero co-culture for 48 h, determined by intracellular NS3 staining by flow cytometer. Data are representative flow cytometry plots **(E)** and quantification **(F)** of *n*=3 individual donors, represented by open squares.

Based on literature suggesting that secretory pathways and vesicles in cell lines are usurped by dengue virus for the purpose of viral transmission (9, 46, 61), we next aimed to assess whether close-contact co-culture was necessary for dengue virus transmission by infected DCs. To accomplish this, we first confirmed that dengue virus was transmitted by primary human DCs to the permissive Vero cell line in a close-contact co-culture experimental setup. To this end, DCs pre-incubated with dengue virus were subsequently extensively washed to remove input virus, and transmission was determined in co-culture with target Vero cells using flow cytometry (**Figure 5A**). Dengue virus was readily transmitted to Vero cells in this close-contact co-culture scenario (**Figures 5B, C**).We next aimed to monitor the dengue virus transmission rate in the absence of close-contact cell culture in order to probe the role of the DC secretome on dengue virus transmission. To this end, DCs pre-incubated with DENV were subsequently extensively washed to remove input virus, and conditioned medium (CM) was subsequently harvested from DENV-infected DCs. Cell-depleted CM was then cultured with the Vero cell line for 48 h, and DENV transmission to Vero cells was analyzed using flow cytometry (**Figure 5D**). Strikingly, we observed robust DENV transmission via DC-derived CM (**Figures 5E, F**), indicating that cell-cell contact is not a prerequisite for viral transmission and pointing towards an alternative cell-free mechanism by which DCs promote DENV transmission to target sites.

### 2.6 DC-derived extracellular vesicles promote dengue virus transmission

**Figure 6:**
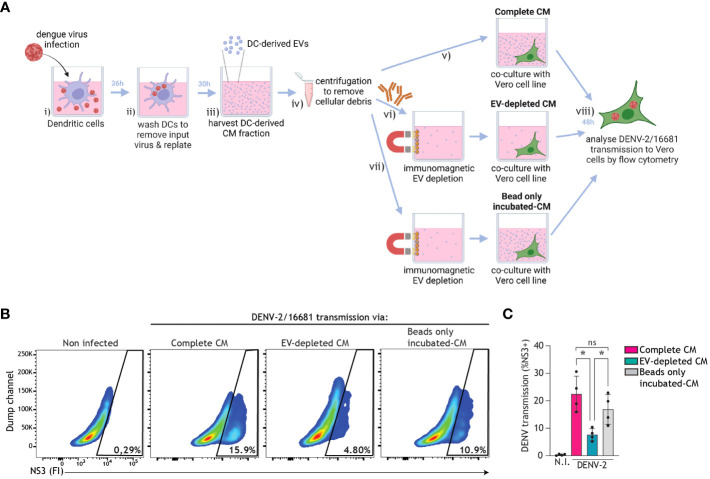
EVs derived from infected DCs facilitate dengue virus transmission. **(A)** Graphical representation of the experimental strategy utilized to determine dengue virus transmission via DC-derived EVs in **(B, C)**. DCs were infected with DENV-2/16681, or mock infected, for 36 h. DCs were then extensively washed to remove input virus, and replated for 30 h. CM was then harvested, centrifuged to remove remaining DCs and cellular debris, and thereafter either left intact, or subjected to positive immunomagnetic depletion of CD9/CD81/CD63+ EVs, or incubated with magnetic beads only. Subsequently, DC-derived complete CM, or EV-depleted CM, or beads only incubated-CM was co-cultured for 48h with the Vero cell line. DENV-2/16681 transmission to Vero cells was determined by intracellular NS3 staining and flow cytometry analysis. **(B, C)** Data are representative flow cytometry plots **(B)** and quantification **(C)** of *n*=4 donors, represented by closed circles. ns, non-significant; *P < 0.05, ANOVA.

In order to assess whether extracellular vesicles (EVs) released into CM by DCs contribute to DENV transmission, we harvested CM from DENV-infected DCs that was either left intact or depleted from EVs using positive immunomagnetic selection of CD9/CD81/CD63+ EVs. Complete CM or EV-depleted CM was then cultured with the Vero cell line for 48 h, and DENV transmission to Vero cells was analysed using flow cytometry (**Figure 6A**). DC-derived CM incubated with magnetic beads only was used as an additional control for potential non-specific binding during the immunomagnetic isolation. Strikingly, DENV transmission by complete CM or beads-only incubated-CM was consistently higher than that by EV-depleted CM (**Figures 6B, C**). Furthermore, TCID50 measurements confirmed the decreased infectious virus levels in the supernatant after EV depletion (TCID50 = 2.49-10.2*10^5^) as compared to prior EV depletion (TCID50 = 5.53-5.68*10^6^). These findings suggest that DC-derived EVs support DENV transmission.

### 2.7 Phenotypical characterization of EVs secreted by DCs reveals an EV subset that co-expresses the autophagy-associated LC3 molecule and contributes to DENV transmission

**Figure 7:**
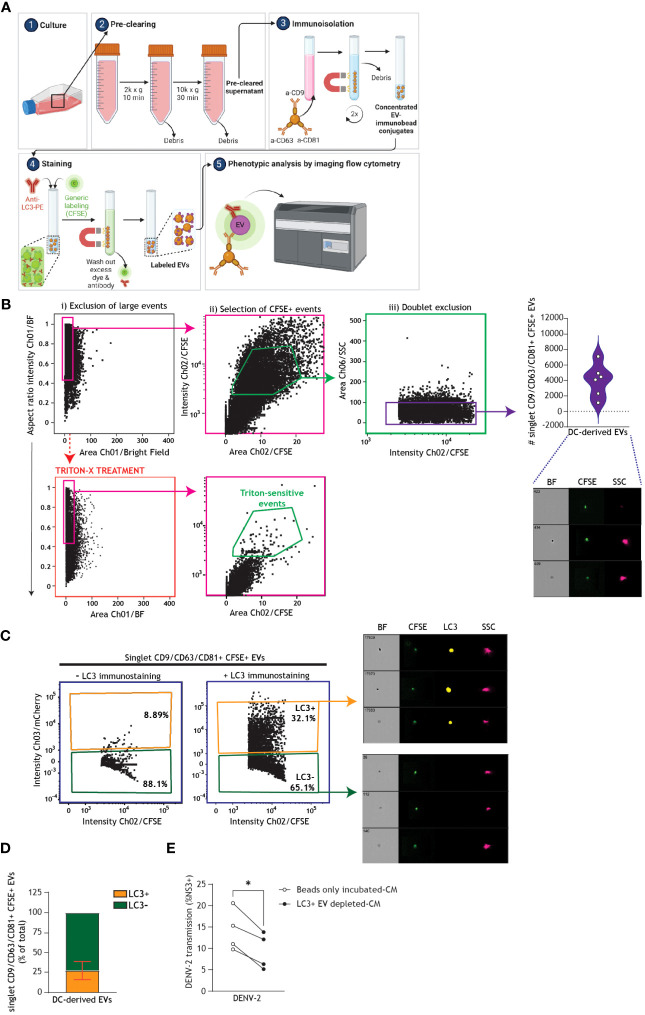
Extracellular autophagy vesicles released by human DCs support dengue virus transmission. **(A)** Graphical representation of the experimental protocol and rationale utilized for phenotypic characterization and quantification of DC-derived CD9/CD81/CD63+ singlet EVs using imaging flow cytometry, as presented in **(B-D)**. Following overnight culture of DCs in FCS-depleted culture medium supplemented with bafilomycin A1 (100 nM), conditioned medium (CM) was collected and pre-cleared by serial centrifugation. EVs were immunomagnetically isolated using a pan-extracellular vesicle positive selection kit, and thereafter stained and analysed by imaging flow cytometry (62). General membrane labelling was performed using carboxyfluorescein succinimidyl ester (CFSE; 300 µM). Samples were additionally stained with anti-LC3 antibody, or left only CFSE-stained as a control, and thereafter EVs were washed twice more to remove residual dye and antibody prior to imaging flow cytometry analysis. **(B)** Gating strategy for analyses of DC-derived EVs, following immunomagnetic isolation and membrane labelling as outlined in **(A)**. To reduce swarming or coincident event detection (“large events”) during individual EV analysis, EV samples were serially diluted in PBS to determine an operational range for analysis by imaging flow cytometry, at which event rate increase was proportional to sample dilution [89].To confirm selection for membrane-bound particles, sub-samples were lysed with 1% Triton X-100 for 60 minutes (63, 64). Closed circles represent the total number of CD9/CD81/CD63+ CFSE+ singlet EVs measured from *n*=6 donors. **(C)** Representative imaging flow cytometry plots and **(D)** quantification of a subset of DC-derived CD9/CD81/CD63+ CFSE+ singlet EVs that co-express LC3, determined by immunostaining followed by imaging flow cytometry analysis. **(D)** Data represent the percentage of LC3+ (orange) versus LC3- (green) CD9/CD81/CD63+ CFSE+ singlet EVs measured in samples derived from *n*=6 DC donors. **(E)** DCs were infected with DENV-2/16681 for 36 h. DCs were then extensively washed to remove input virus, and replated for 30 h. CM was then harvested, centrifuged to remove remaining DCs and cellular debris, and thereafter either subjected to positive immunomagnetic depletion of LC3+ EVs (LC3+ EVs-depleted CM), or incubated with magnetic beads only (beads only incubated-CM). Subsequently, each CM sample was co-cultured for 48h with the Vero cell line and DENV-2/16681 transmission to Vero cells was determined by intracellular NS3 staining and flow cytometry analysis. *n* = 4 donors, *P < 0.05, Paired *t*-test.

Next, we undertook positive immunomagnetic isolation of EVs expressing CD9, CD81, or CD63 from the supernatant of bafilomycin A1-treated DCs and used an imaging flow cytometry-based methodology to assess the interplay between autophagy and EVs biogenesis in human DCs (**Figure 7A**). To this end, DC-derived CM was collected and subjected to serial centrifugation steps to pre-clear it of cellular debris. EVs within the pre-cleared CM were then labeled with tetrameric antibody complexes recognizing CD9, CD81, and CD63, alongside magnetic particles. Labeled EVs were then magnetically separated (63, 64) from unwanted biofluid and debris, washed, and stained with general membrane dye CFSE to select for membrane-bound particles. CFSE-positive events could then be discriminated from remaining cellular debris or unbound magnetic beads via exclusion of large/coincident events followed by doublet exclusion (**Figure 7B**) (62, 65–67). Gating of singlet CFSE+ membrane-bound particles was confirmed by treating a subset of samples with Triton-X detergent (**Figure 7B**) (67, 68). Singlet CD9/CD81/CD63+ EVs were robustly secreted across six DC donors (**Figure 7B**). Furthermore, RT-qPCR analyses confirmed the presence of viral RNA in isolated EV samples from DENV-infected DCs, as compared to uninfected DCs (**Supplementary Figure 3**). Previous reports in human cell lines have demonstrated that LC3+ vesicles can be trafficked for secretion (46, 69). In line with these reports, using the standardized autophagy reporter human cell line (26, 50, 70), we observed that fluorescently-labeled intracellular LC3+ vesicles can be released extracellularly (**Supplementary Figure 4**). Markedly, subsequent immunophenotypical characterization of secreted vesicles by human DCs demonstrated that an average of 26% of the total population of CD9/CD81/CD63+ DC-derived EVs co-expressed the autophagy-associated LC3 marker (**Figures 7C, D**). These data underpin the intersection of autophagy machinery with secretory pathways and uncover a LC3+ EV subset released by human primary DCs. To determine whether LC3+ EVs released by DCs contribute to DENV dissemination, we harvested CM from DENV-infected DCs, which was either incubated with magnetic beads (Beads-only incubated CM) or depleted of LC3+ EVs (LC3+EV-depleted CM) using positive immunomagnetic isolation, and subsequently cultured with the Vero cell line for 48 h (**Figure 7E**). Notably, depletion of LC3+ EVs from DC-derived CM reduced DC-mediated DENV transmission. Taken together, these data suggest that DENV hijacks host cell secretory pathways leading up to the release of EVs, including autophagy-associated vesicles, by human DCs to promote viral dissemination.

## 3 Discussion

Despite recent progress in prophylactic strategies, dengue virus remains a major threat to global public health. Historically, vaccine development for DENV has been fraught, due to the danger of vaccine-elicited antibodies leading to antibody-dependent enhancement in seronegative patients (2, 71). The first approved vaccine protective against dengue virus was a live-attenuated vaccine developed by Sanofi Pasteur, termed CYD-TDV (Dengvaxia^®^), which is currently recommended only for individuals who have previously been infected with dengue virus and are already seropositive (2, 72). More recently, a second live attenuated vaccine developed by Takeda, termed Qdenga, was demonstrated to be safe regardless of serostatus and is now approved for use in Europe (73). Although these vaccines represent breakthrough progress in prevention of dengue virus infections, there are still no specific antiviral drugs against DENV, and the cornerstone of post-infection treatment remains simply supportive therapies such as fluid management (1, 72). In order to develop novel antiviral therapeutics, including host-directed therapeutics – which tend to be functional across different viral serotypes and relatively impervious to viral resistance mutations (26, 50, 74) – improved understanding of the host pathways targeted by dengue virus during early infection of its major human target cells is imperative. In particular, DCs represent a pertinent therapeutic cellular target due to their key positioning at the intersection of innate and adaptive immunity and their documented secretion of cytokines, chemokines, and interferons upon DENV exposure (10–14, 49).

This study underpins the pro-viral roles of autophagy pathways and vesicles in dengue virus infection of primary human dendritic cells. Our data demonstrates that host ULK1/ATG5/ATG16L1-dependent autophagy mechanisms support the establishment of DENV-2 infection of DCs (**Figures 1D–I**, **Supplementary Figures 1A–D**). Furthermore, we show that DENV-2 triggers autophagosome biogenesis and that DENV-2 replication intermediates are associated with LC3+ autophagy vesicles in productively infected DCs (**Figures 2A–D**). These findings are in line with previous data demonstrating that ATG5 is integral in DENV-2-triggered autophagosome formation (45) and that ATG5-deficient Huh7 are less able to transmit dengue virus to target cells (46). The relatively higher susceptibility rate of DENV infection in *ATG16L1* rs6861 (TT) genotyped DCs (**Figures 4B, C**), endowed with intrinsically higher numbers of autophagosomes (**Figure 4A**) (52), aligns with previous reports in cell lines and mice showing that dengue virus-induced autophagosomes accumulation promotes viral replication (45, 75). Taken together, these findings highlight that autophagosome biogenesis represents a host determinant of DENV replication in human DCs, and hint towards possible genetic underpinnings of differential viral burden. Future studies are required to establish casual relationships between autophagy phenotypes and DENV susceptibility in these genotyped DCs as the function of the *ATG16L1* rs6861 genetic variant is yet unknown.

Previous reports in cell lines have suggested that dengue virus obstructs the later degradative stages of autophagy in order to avoid p62-mediated autophagic degradation (41, 42). Our data underscores that dengue virus institutes a block in autophagy flux in primary DCs, as evident by accumulation of LC3-II and p62 in the presence of dengue virus (**Figures 3A–E**) but a lack of LC3-II accumulation upon concomitant dengue virus infection and bafilomycin treatment (**Supplementary Figures 2A, B**). In addition, we demonstrate that autophagy flux impairment is dependent on active viral replication (**Figure 3C**, **Supplementary Figure 2C**). Blocking autophagy flux to avoid selective targeting for degradation, often in a cell-specific manner, is a common immune evasion strategy across many clinically-relevant viruses, such as SARS-CoV-2 and picornaviruses (26, 51, 53, 76). Indeed, picornaviruses in particular subvert autophagy in a similar manner to that of dengue virus, as uncovered by our study herein – namely, in that they rely on early autophagy machinery for establishment of infection, and subsequently institute a block in autophagy flux to promote virus replication (76). It is now well-established that there are two alternative fates for autophagy vesicles: once generated, they may be destined for either the degradative autophagy or the secretory autophagy pathway (25, 29, 77, 78). Recent research has highlighted that differential molecules govern secretory versus degradative autophagy (28, 37, 79), and although additional studies are necessary to fully characterize the cross-talk between degradative and secretory autophagy pathways, previous data have hinted that a blockage of autophagy flux could result in re-routing of autophagy vesicles towards secretion rather than degradation (37). Importantly, our data demonstrates that dengue virus is readily transmitted by primary human DCs via not only direct cellular contact (**Figures 5A–C**), but also via a cell-free, EV-facilitated mechanism (**Figures 5D–F**, **6A–C**). EVs have recently been highlighted as important players in viral transmission and dissemination across many different viral families – both in promoting and suppressing viral spread (46, 61, 69, 80–83). Previous reports have shown that EVs secreted by DCs can carry host microRNAs or viral components for the purpose of immune modulation, which may serve to counteract viral infection (61, 83). Specifically in regards to DENV, EVs derived from DCs treated with interferon alpha were demonstrated previously to exert a protective effect against DENV infection of other cells (61). On the other hand, in the case of not only dengue virus but also SARS-CoV-2, HIV-1, and the encephalomyocarditis picornavirus, EVs secreted from human cells are exploited to transmit infectious virus (46, 61, 69, 81, 82).

Our study demonstrates for the first time that primary human cells release LC3+ vesicles extracellularly, and that this LC3+ EV subset represents approximately one quarter of the CD63/CD9/CD81+ EVs secreted by human DCs (**Figure 7D**). Interestingly, previous studies have reported that autophagy-deficient Huh7 cells produced fewer LC3 co-expressing EVs upon infection with DENV, pointing towards a role for secretory autophagy in DENV-induced EV secretion (46). Consistent with previous reports demonstrating EV-mediated DENV dissemination from mosquito and human cell lines (9, 46–48), herein we demonstrate that DENV-2 transmission to target cells is reduced upon depletion of CD63/CD9/CD81+ EVs, as well as LC3+ EVs, suggesting a role for DC-derived EV populations in DENV-2 transmission (**Figures 6A–C**, **7D, E**). In addition, we confirmed the capacity of DC-derived EVs to carry DENV-2 genomic material (**Supplementary Figures 3A, B**). Recent data highlighted that the encephalomyocarditis picornavirus stimulates an increase in autophagosome generation in the human HeLa R19 cell line, and that those autophagosomes are subsequently routed to secretion to result in release of infectious extracellular autophagy vesicles (69). Our data builds upon previous descriptions of secretory autophagy as an alternative delivery mechanism for intracellular autophagosome-containing cargo to the extracellular milieu (25, 29, 30, 69, 84), and sets the stage for future further characterization of EV subpopulations. EV-mediated transmission serves as a stealthy mechanism by which viruses can escape neutralization by antibodies and thereby likely accomplish longer range dissemination through patient bloodstreams or lymphatic systems to reach distal target cells (69, 81, 82, 85, 86). Follow-up studies with sorted subpopulations of DC-derived EVs in combination with transcriptomics and/or proteomics approaches should elucidate the specific roles of LC3+ EVs in dengue virus infection and disease pathogenesis.

In conclusion, our data provides robust evidence that DENV-2/16681 concomitantly exploits early stages of autophagy and blocks the late degradative stages of autophagy leading to accumulation of autophagosomes in productively infected primary human DCs. Furthermore, we demonstrate that primary human DCs express LC3+ vesicles both intra- and extracellularly. This study suggests that DENV-2 replication intermediates are associated with LC3+ autophagosomes, and that higher abundance of autophagosomes is likely associated with increased DENV susceptibility in DCs, demonstrating that autophagosome biogenesis is a key determinant of dengue virus susceptibility. Finally, we also present evidence that CD9/CD81/CD63+ EVs, including LC3+ EVs subset, represents a novel cell-free mechanism for dengue virus dissemination following infection of DCs. Looking forward, we envision that interfering in the molecular mechanisms underlying autophagosome generation and/or secretion of infectious EVs could underpin novel host-directed antiviral strategies for limiting dengue virus dissemination and pathogenesis.

## 4 Materials and methods

### 4.1 Dendritic cell isolation

Monocyte-derived DCs were generated either by gradient centrifugation as previously described (26, 27, 52), or using positive magnetic-activated cell-sorting for CD14+ monocytes according to the manufacturers’ instructions (Miltenyi). Briefly, PBMCs were isolated from buffy coats of healthy donors (Sanquin) using a Lymphoprep (Axis-Shield) gradient. Subsequently, monocytes were enriched using either a Percoll (Amersham Biosciences) gradient step or by positive immunomagnetic selection with CD14 Microbeads (Miltenyi, 130-050-201). Isolated monocytes were then differentiated into immature DCs over 6 days RPMI 1640 containing 10% FCS, penicillin/streptomycin (10 U/ml and 10 μg/ml, respectively; Invitrogen), and 2 mM L-glutamine (Lonza), and supplemented with 500 U/ml IL-4 (Invitrogen) and 800 U/ml GM-CSF (Invitrogen). This protocol consistently generates high-purity immature DCs expressing typical markers DC-SIGN, CD11c, and CD1a, as measured by flow cytometry (26).

### 4.2 Genotyping

Genotyping to determine the *ATG16L1* rs6861(TT and CC) genetic variants was performed on blood samples from healthy donors (Sanquin Blood Supply Foundation) using the TaqMan Sample-SNP kit (ThermoFisher) according to manufacturer instructions, as previously described (52).

### 4.3 Cell lines

The U87 cell line stably expressing CD4 and wild-type CCR5 co-receptor was obtained through the NIH AIDS Reagent Program, Division of AIDS, NIAID, NIH: U87 CD4^+^CCR5^+^ cells, ARP-4035, contributed by Drs. HongKui Deng and Dan Littman (26, 27, 87). Autophagy reporter cells were generated via retroviral transduction of U87.CD4.CCR5 with pBABE-mCherry-GFP-LC3 [Addgene 22418; gift from Prof. Jayanta Debnath (88)], as described previously (26, 70), hereafter referred to as U87.LC3-mCherry-GFP cells. The U87.LC3-mCherry-GFP cell line was maintained in Iscoves Modified Dulbecco’s Medium (IMDM, Thermo Fischer Scientific, USA) supplemented with 10% FCS and penicillin/streptomycin (10 U/ml and 10 μg/ml, respectively; Invitrogen). In this cell line, LC3 molecules are tandem tagged with both mCherry and GFP, resulting in dual fluorescence of LC3+ vesicles. Because GFP is more acid-sensitive than mCherry, a reduction in GFP signal is indicative of fusion of autophagosomes and lysosomes, i.e. autophagy flux (**Supplementary Figures 4A, B**). Here, we utilised the mCherry signal of EVs derived from U87.LC3-mCherry-GFP cells to signify LC3 expression, in combination with CFSE general membrane staining to select for only membrane-bound EVs, as was performed with primary human DCs (**Supplementary Figure 4C**). The Vero African green monkey cell line (ATCC CLL-81) was a gift from Dr. Julia Eder and Prof. Teunis Geijtenbeek of Amsterdam UMC. Vero cells were maintained in Dulbecco’s Modified Eagle Medium (DMEM, Gibco) supplemented with 10% FCS, 2mM L-glutamine (Lonza), penicillin/streptomycin (10 U/ml and 10 μg/ml, respectively; Invitrogen), and non-essential amino acids (Sanbio).

### 4.4 Intracellular staining of p62

Cells were fixed in 4% paraformaldehyde (PFA; Electron Microscopy Sciences) and subsequently permeabilized with PBS containing 0.5% saponin and 1% bovine serum albumin (BSA; both Sigma). Permeabilization was followed by staining with either mouse anti-p62 (IgG2a, 5 μg/mL, Abcam ab56416) or mouse IgG2a isotype control (5 μg/mL, eBioscience 14-4724-85), and thereafter staining with goat anti-mouse IgG2a-Alexa Fluor 488 (Invitrogen A11029). Intracellular p62 levels were quantified using flow cytometry (FACSCanto II, BD Biosciences).

### 4.5 Intracellular staining of autophagy vesicles

Cells were either left untreated or pre-treated with bafilomycin A1 (*In vivo*gen tlrl-baf, 50nM) for 4h followed by infection with DENV-2/16681 (MOI=6) for 48h. Quantification of intracellular LC3 II levels by saponin extraction was performed as described before (26, 27, 52, 58). In assays using dengue-infected DCs, intracellular staining of LC3-II was performed using a two-step staining with anti-human LC3 (4E12; MBL Life Science) followed by goat anti-mouse IgG1 (AF488; Invitrogen A-21121). In assays using non-infected genotyped DCs, intracellular staining of LC3-II was performed using a single-step staining with anti-LC3 monoclonal antibody (4E12; MBL Life Science) directly conjugated to AF488 using the Lightning-Link^®^ Rapid Antibody Labeling kit (Expedeon), according to the manufacturer’s instructions. Intracellular LC3-II levels were quantified using flow cytometry (FACSCanto II, BD Biosciences).

### 4.6 Immunoblotting for LC3

Cells were either left untreated, treated with bafilomycin A1 (50nM), or concomitantly treated with bafilomycin A1 (50nM) and infected with DENV-2/16681 (MOI = 6) for 48h. Whole-cell extracts were prepared using RIPA lysis buffer supplemented with protease inhibitors (9806S; Cell Signaling). 20–30 μg of extract were resolved by SDS–PAGE (15%) and immunoblotted with LC3 (2G6; Nanotools) and β-actin (sc-81178; Santa Cruz) antibodies, followed by incubation with HRP-conjugated secondary rabbit-anti-mouse antibody (P0260; Dako) and luminol-based enhanced chemiluminescence (ECL) detection (34075; Thermo Scientific) using ImageQuant LAS4000. Quantitative analyses were performed using Thermo Fisher Scientific MYImageAnalysis 2.0 software, by normalizing median band intensity of LC3-II to respective median band intensity of β-actin (protein loading control) and relative lane intensity was obtained by setting untreated DCs to 1.

### 4.7 Confocal microscopy

DCs seeded on 18-well microslides (Ibidi, 81811) coated with poly-L-lysine (Santa Cruz BioTechnology, SC286-689) and infected with DENV-2/16681 for 48 h, with or without treatment with flavivirus replication inhibitor SDM25N (10 µM, Tocris Bioscience), were fixed in 4% paraformaldehyde (PFA; Electron Microscopy Sciences) for at least 30 minutes at RT and then extensively washed with PBS. Samples were permeabilized with 0.5% Triton-X100 for 10 minutes, and subsequently blocked with Fish serum blocking buffer (Thermofisher) for 1h at RT. Cells were incubated overnight at 4°C with primary antibodies Mouse IgG2a anti-dsRNA (clone J2, Jena BioSciences, RNT-SCI-10010, diluted 1:800) or Mouse IgG1 anti-LC3 (clone 4E12, MBL, M152-3, diluted 1:100) in Fish serum blocking buffer. This was followed by secondary antibodies goat anti-mouse IgG2a-Alexa 488 (Invitrogen A11029) and goat anti-mouse IgG1-Alexa 647 (Invitrogen A-21240) diluted at 1:400, and the probe Phalloidin CruzFluor™-488 (Santa Cruz Biotechnology) diluted at 1:1500 in Fish serum blocking buffer for 1h at RT. Nuclei were stained with 300nM DAPI (Invitrogen). Samples were imaged on a Leica TCSS SP8 X mounted on a Leica DMI6000 and analysed using LAS X. Image brightness was adjusted using Adobe Photoshop for visualization clarity; all analyses were performed on raw images. Quantitative analyses were performed by measuring the mean grey value with ImageJ software (version 150.i) on 10 fields of view per donor. Pearson’s correlation coefficient was calculated using LAS X software on 10 ROIs per DC donor.

### 4.8 RNA interference

RNA interference was performed using the Neon Transfection System according to manufacturer’s protocol (Thermo Fisher) and as previously published [26,46]. Primary human DCs were transfected with short interfering (si) SMARTpool RNAs (Dharmacon): siATG5 (M-004374-04), siATG16 (M-021033-02), siATG13 (M-020765-01-0010), siFIP200 (M-021117-01-0005), or siNon-Target as a control (D-001206-13) at end concentrations of 0.5µM siRNA per million of cells. Transfected DCs, as well as non-transfected cells as an additional control, were seeded in 96-well plates in RPMI 1640 containing 10% FCS and 2 mM L-glutamine (Lonza), and supplemented with 500 U/ml IL-4 (Invitrogen) and 800 U/ml GM-CSF (Invitrogen), without antibiotics. After 24 hours, cells were washed, counted, and replated. Silencing of expression of target genes in DCs was confirmed by quantitative real-time PCR.

### 4.9 RNA isolation and quantitative real-time PCRs

mRNA was isolated from DCs and EVs with mRNA Catcher™ PLUS Purification Kit (Thermofisher) and cDNA was synthesized with a reverse-transcriptase kit (Promega). For real-time PCR analysis, PCR amplification was performed in the presence of SYBR green for 40 cycles in a 7500 Fast Realtime PCR System (ABI). Specific primers for host genes were designed with Primer Express 2.0 (Applied Biosystems) and DENV-2 RNA Primers were designed with Merck custom DNA Oligo (**Table 1**). The cycling threshold (*C*_t_) value is defined as the number of PCR cycles in which the fluorescence signal exceeds the detection threshold value. For DCs, expression of target genes was normalized to GAPDH (N_t_ = 2^Ct(GAPDH)–Ct(target)^) and relative mRNA expression in siRNA transfected DCs was obtained by setting siNon-Target control to 1 for each donor. *C*_t_ values and melting curves profiles of viral RNA amplicon are shown for isolated EVs derived from DENV-infected DCs. Undetected amplicon in Non-infected DCs was set *C*_t_=40 (89, 90).

**Table 1:** mRNA expression primer sequences.

Primer	Sequence
**GAPDH *forward***	CCATGTTCGTCATGGGTGTG
**GAPDH *reverse***	GGTGCTAAGCAGTTGGTGGTG
**ATG5 *forward***	TCATTCAGAAGCTGTTTCGTCC
**ATG5 *reverse***	CCCCATCTTCAGGATCAATAGC
**ATG16L1 *forward***	TGCTCCCGTGATGACTTGC
**ATG16L1 *reverse***	CAACTCTGGTCCAGTCAGAGCC
**ATG13 *forward***	TGTCCAAGTGATTGTCCAGGC
**ATG13 *reverse***	AACTCCCGATAGAAGGTCCCC
**FIP200 *forward***	ACTGGTGCTCTCTCCTGATATGCC
**FIP200 *reverse***	CGACACATGTTCCACTGACTTGG
**DENV-2 RNA *forward***	AAGGTGAGATGAAGCTGTAGTCTC
**DENV-2 RNA *reverse***	CATTCCATTTTCTGGCGTTCT

### 4.10 Dengue virus production

DENV-2/16681 confirmed by Sanger Sequencing was kindly provided by Dr. Brandy Russell of the Arbovirus Reference collection (ARC) from the Centers for Disease Control and Prevention (CDC; Fort Collins, CO, USA). DENV-2/16681 was amplified in the C6/36 mosquito cell line as described previously (13, 49). Briefly, 80% confluent C6/36 cells cultured in RPMI 1640 supplemented with 2% FCS, penicillin/streptomycin (10 U/ml and 10 μg/ml, respectively; Invitrogen), and 2mM L-glutamine (Lonza) were exposed to DENV-2/16681 for 7-9 days. Thereafter, supernatant derived from infected C6/36 cells was harvested and subsequently cleared from cellular debris by centrifugation followed by filtration at 0.2 µM. Virus-containing supernatant was stored at -80°C, and viral titres were determined by TCID50 measurements in Vero cells as previously described (91).

### 4.11 Dengue virus infection

Primary human DCs were infected with DENV at MOI=6, and harvested after 48 hours. Dengue virus infection using NS3 immunostaining was quantified using flow cytometry as described before (FACSCanto II, BD Biosciences) (13, 49). Infected cells were fixed in 4% PFA (Electron Microscopy Sciences) and subsequently permeabilized with PBS containing 0.5% saponin and 1% bovine serum albumin (BSA; both Sigma). Cells were stained with rabbit anti-NS3 (1:400 SAB2700181, Sigma) followed by followed by staining with AF647-conjugated anti-rabbit (Invitrogen A-31573). Measurement of productive infection was confirmed by concurrent treatment of cells with flavivirus replication inhibitor SDM25N (10 µM, Tocris Bioscience).

### 4.12 Dengue virus transmission

Primary human DCs were infected with DENV-2 at MOI=6, and subsequently washed extensively after 36 hours to remove input virus. 96 h p.i., infected samples were centrifuged at 1500 rpm and either the cell-enriched fraction (containing DENV-infected DCs) or cell-depleted fraction (containing DC-derived condition medium, CM) were harvested. DENV-infected DCs, or CM (either complete, upon EV-depletion, or upon incubation with magnetic beads only) were then co-cultured with the susceptible Vero cell line for 48 hours, after which cells were fixed in 4% PFA (Electron Microscopy Sciences) and subsequently permeabilized with PBS containing 0.5% saponin and 1% bovine serum albumin (BSA; both Sigma). Vero cells were stained with rabbit anti-NS3 (1:400 SAB2700181, Sigma) followed by staining with AF647-conjugated anti-rabbit (Invitrogen A-31573), in combination with mouse anti-CD11c-PECy7 clone B-ly6 (BD 561356) to discriminate the percentage of NS3+CD11c^-^ infected Vero cells. Dengue virus transmission was quantified using flow cytometry (FACSCanto II, BD Biosciences).

### 4.13 Extracellular vesicle isolation and characterization

Extracellular vesicles were isolated using EasySep Human Extracellular Vesicle Positive Selection kits (StemCell, cat# 17891, 17892, 17894, 17895) according to the manufacturers’ instructions. Briefly, following overnight culture in FCS-depleted culture medium with or without bafilomycin A1 (100 nM), cell supernatant derived from either U87.LC3-mCherry-GFP cells or DCs was collected and pre-cleared by serial centrifugation (10 minutes at 2000x *g* followed by 30 minutes at 5000x *g*). Pre-cleared supernatant was then either stored at -20°C for future use, or immediately incubated with the EasySep selection cocktail containing antibodies selecting for classical EV markers human CD9 and/or CD63 and/or CD81, followed by incubation with magnetic RapidSpheres for positive immunomagnetic isolation. After rigorous mixing, EVs bound to immunomagnetic beads were washed twice, and thereafter stained and analysed by imaging flow cytometry (62). General membrane labelling was performed using carboxyfluorescein succinimidyl ester (CFSE; 300 µM, Invitrogen, C1157). Additionally, samples were stained with anti-LC3B-PE antibody (1:50 BioTechne, NB600-1384PE), or left only CFSE-stained as a control, and thereafter EVs were washed twice more to remove residual dye and antibody. To exclude swarming or coincident event detection during individual EV analysis, EV samples were serially diluted in PBS to determine an operational range for analysis by imaging flow cytometry, at which event rate increase was proportional to sample dilution (92). To confirm selection for membrane-bound particles, sub-samples were lysed with 1% Triton X-100 for 60 minutes (67, 68) prior to EV phenotypic characterization and quantification (62, 65) using the ImageStream Mk II (Amnis).

### 4.14 Extracellular vesicles depletion

CM derived from DENV-infected DCs was subjected to EV depletion using the EasySep Human Pan-Extracellular Vesicle Positive Selection kit (StemCell, cat# 17891) or EasySep Extracellular Vesicle PE Positive Selection Kit (StemCell, cat# 100-0812). Cell supernatant was first collected and pre-cleared by serial centrifugation (10 minutes at 2000x *g* followed by 30 minutes at 10000x *g*). Pre-cleared supernatant was then either immediately incubated with the EasySep selection cocktail containing antibodies selecting for classical EV markers human CD9, CD63, and CD81, or incubated with 1µg/mL anti-LC3-PE (BioTechne, NB600-1384PE) followed by EasySep selection PE positive selection cocktail. Samples were subsequently incubated with magnetic RapidSpheres for positive immunomagnetic isolation. Incubation of pre-cleared supernatant with magnetic RapidSpheres was used as negative control (beads only-incubated CM). After rigorous mixing and incubation, CD9/CD81/CD63+ EVs or LC3+ EVs were left bound to the immunomagnetic beads and EV-depleted CM was collected. EV-depleted CM, Beads only-incubated CM, or complete CM (not subjected to EV depletion as a control), derived from DENV-infected DCs was then co-cultured with Vero cells. Dengue virus infection of Vero cells was assessed by intracellular staining of viral protein NS3 by flow cytometry analyses.

### 4.15 Statistical analyses

Data were analysed using FlowJo, LLC, version 10 (Treestar), IDEAS version 6.3 (Amnis), and/or GraphPad Prism 9™ (Graphpad Software, Inc.). Two-tailed, parametric students *t*-tests were performed for paired observations, and a two-tailed, unpaired *t*-test was performed for independent observations. Multiple comparisons were analysed by one-way ANOVA. For independent observation in which data is shown in relative, a one-sample *t*-test was used to compare fold changes in experimental conditions to the hypothetical population mean of 1, with untreated or uninfected samples set to equal (24, 57).

## Data availability statement

The original contributions presented in the study are included in the article/**Supplementary Material**. Further inquiries can be directed to the corresponding author.

## Ethics statement

Buffy coats derived from blood donations (Sanquin blood bank, the Netherlands) were obtained with approval of the Medical Ethics Review Committee of the Amsterdam UMC. Buffy coats were handled in accordance with the relevant guidelines and regulations, as stated in the Amsterdam UMC Research Code. Use of buffy coats is not subjected to informed consent according to the Medical Research Involving Human Subjects Act and the Medical Ethics Review Committee of Amsterdam UMC.

## Author contributions

AC: Conceptualization, Data curation, Formal analysis, Funding acquisition, Investigation, Methodology, Visualization, Writing – original draft, Writing – review & editing. AR: Conceptualization, Data curation, Formal analysis, Investigation, Visualization, Writing – review & editing. KP: Formal analysis, Investigation, Writing – review & editing. TE: Conceptualization, Methodology, Validation, Writing – review & editing. SP: Formal analysis, Investigation, Methodology, Writing – review & editing. RS: Formal analysis, Investigation, Validation, Writing – review & editing. CR: Conceptualization, Funding acquisition, Investigation, Project administration, Resources, Supervision, Validation, Writing – original draft, Writing – review & editing.

## Supplementary material

Supplementary Figure 1 Ulk1-Atg5-Atg16L1-dependent autophagy machinery facilitates dengue virus infection of human DCs. **(A–D)** Viral infection of DCs upon transfection with siATG5, siATG16L1, siATG13 or siFIP200, or non-targeting control siRNA as control, followed by exposure to DENV-2/16681 for 48 h, determined by intracellular NS3 staining. **(A, B)** Quantification of DENV-2 infection presented on **Figures 1E, H**. **(D)** ATG13 or FIP200 silencing efficiency was determined by real-time PCR. mRNA expression was normalised to GAPDH and set at 1 in cells transfected with control siRNA. Closed circles represent *n*=5 **(A)** *n*=or 4 **(B)** donors measured, **P* < 0.05, student’s *t*-test **(C, D)** Bars represent the mean of *n*=2 donors measured.

Supplementary Figure 2 Lack of autophagy flux in DENV-infected human DCs. **(A, B)** Autophagy activity in primary human DCs either left untreated, treated with bafilomycin A1, or concomitantly treated with bafilomycin A1 (50nM) and infected with DENV-2/16681 for 48 h, determined by intracellular LC3-II levels using saponin extraction (26, 27, 52, 58) **(A)**, or by immunoblotting for LC3 **(B)**. Relative abundance of LC3II determined by normalizing to β-actin. **(A, B)** Data are representative of *n* = 3 donors. **(C)** Intracellular p62 levels in primary human DCs infected with DENV-2/16681 for 48 h, with or without concomitant treatment with flavivirus replication inhibitor SDM25N (10 µM). Data are representative flow cytometry plots of *n* = 2 donors, determined by intracellular p62 staining.

Supplementary Figure 3 Detection of LC3+ EVs released from the human U87.LC3-mCherry-GFP autophagy reporter cell line. **(A)** Graphical representation of the U87.LC3-mCherry-GFP cell line, which is typically utilised for monitoring of autophagy flux (26, 50, 70). LC3 is tandem tagged with both mCherry and GFP, resulting in dual fluorescence of intracellular autophagosomes in both red and green channels. Because GFP is more acid-sensitive than mCherry, a reduction in GFP signal is indicative of fusion of autophagosomes and lysosomes, i.e. autophagy flux. **(B)** Autophagy flux analyses in U87.LC3-mCherry-GFP cells, determined by whole-cell fluorescence measurement using imaging flow cytometry. U87.LC3-mCherry-GFP cells containing primarily autophagosomes that follow the canonical late stages of autophagy and fuse with lysosomes appear red due to the quenching of acid-sensitive GFP upon autophagosome-lysosome fusion, while the more acid-stable mCherry conjugated to LC3 remains bright. U87.LC3-mCherry-GFP cells containing primarily accumulated autophagosomes that do not follow the late degradative stages of autophagy express both green (GFP) and red (mcherry) fluorescence (26, 50, 70). **(C)** Use of the U87.LC3-mCherry-GFP autophagy reporter cell line for detection of extracellular LC3+ vesicles. Following overnight culture of U87.LC3-mCherry-GFP cells in FCS-depleted culture medium, conditioned medium (CM) was collected and pre-cleared by serial centrifugation (10 minutes at 2000x *g* followed by 30 minutes at 5000x *g*). EVs were immunomagnetically isolated using a pan-extracellular vesicle positive selection kit, and thereafter stained and analysed by imaging flow cytometry (62). General membrane labelling was performed using carboxyfluorescein succinimidyl ester (CFSE; 300 µM), and thereafter EVs were washed twice more to remove residual dye and antibody. To reduce swarming or coincident event detection during individual EV analysis, EV samples were serially diluted in PBS to determine an operational range for analysis by imaging flow cytometry (92). LC3 expressed on secreted vesicles are tandem-tagged with both mCherry and GFP. Thus, in combination with CFSE general membrane staining to select for only membrane-bound EVs, the mCherry signal of EVs derived from U87.LC3-mCherry-GFP cells can be utilised to quantify LC3 expression of EVs. Shown are a brief gating strategy and imaging flow cytometry plots utilized to confirm the presence of secreted CFSE+ U87.LC3-mCherry-GFP cell line-derived CD9/CD81/CD63+ singlet EVs, including their surface expression of LC3 (mCherry).

Supplementary Figure 4 DC-derived EVs harbour DENV-2 RNA. **(A, B)** Detection of DENV-2 RNA in DC-derived EVs, determined by quantitative real-time PCR. DCs were infected with DENV-2/16681 for 48 h. DCs were then extensively washed to remove input virus, and replated for 30 h. CM was then harvested, centrifuged to remove remaining DCs and cellular debris, and thereafter subjected to positive immunomagnetic selection of CD9/CD81/CD63+EVs. **(A)** Cycle threshold (Ct) values of viral RNA in isolated CD9/CD81/CD63+ EVs derived from either DENV-infected DCs or non-infected DCs. **(B)** Representative profiles of the melting curves of viral RNA amplicon in DENV-infected DCs (green) or isolated CD9/CD81/CD63+ EVs from DENV-infected DCs (fuchsia) or non-template amplification control (orange). **(A, B)** Data represents *n*=4 donors, ***P* < 0.01, student’s *t*-test.
