# Supplementary figures and images for: Dengue virus exploits autophagy vesicles and secretory pathways to promote transmission by human dendritic cells
Source: Front Immunol. 2024 May 28;15:1260439. doi: 10.3389/fimmu.2024.1260439 (PMC11165123; doi:10.3389/fimmu.2024.1260439)

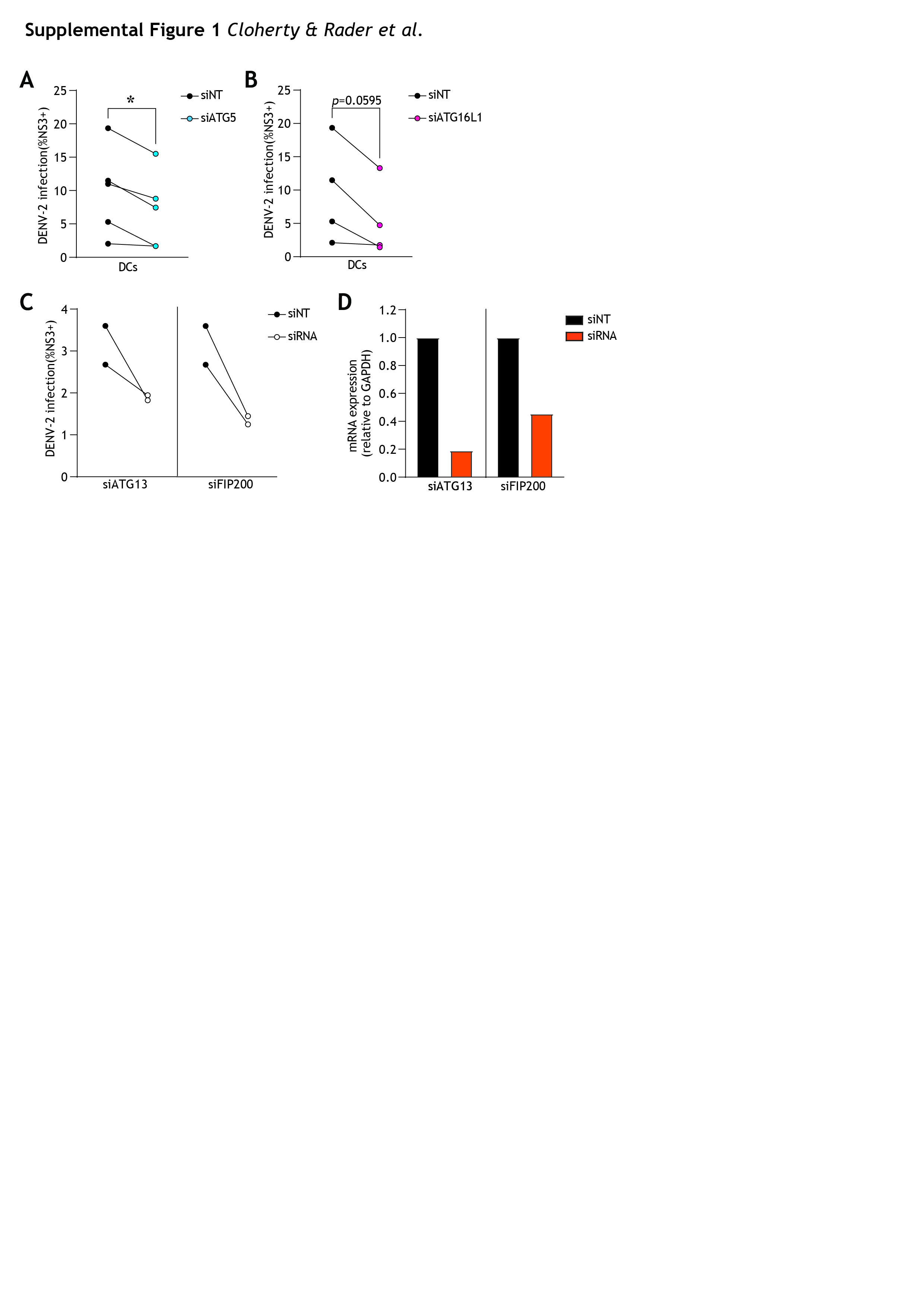

Supplement: Supplementary Figure 1 — Ulk1-Atg5-Atg16L1-dependent autophagy machinery facilitates dengue virus infection of human DCs. (A–D) Viral infection of DCs upon transfection with siATG5, siATG16L1, siATG13 or siFIP200, or non-targeting control siRNA as control, followed by exposure to DENV-2/16681 for 48 h, determined by intracellular NS3 staining. (A, B) Quantification of DENV-2 infection presented on Figures 1E, H . (D) ATG13 or FIP200 silencing efficiency was determined by real-time PCR. mRNA expression was normalised to GAPDH and set at 1 in cells transfected with control siRNA. Closed circles represent n=5 (A) n=or 4 (B) donors measured, *P < 0.05, student’s t-test (C, D) Bars represent the mean of n=2 donors measured. [file Image_1.jpeg]

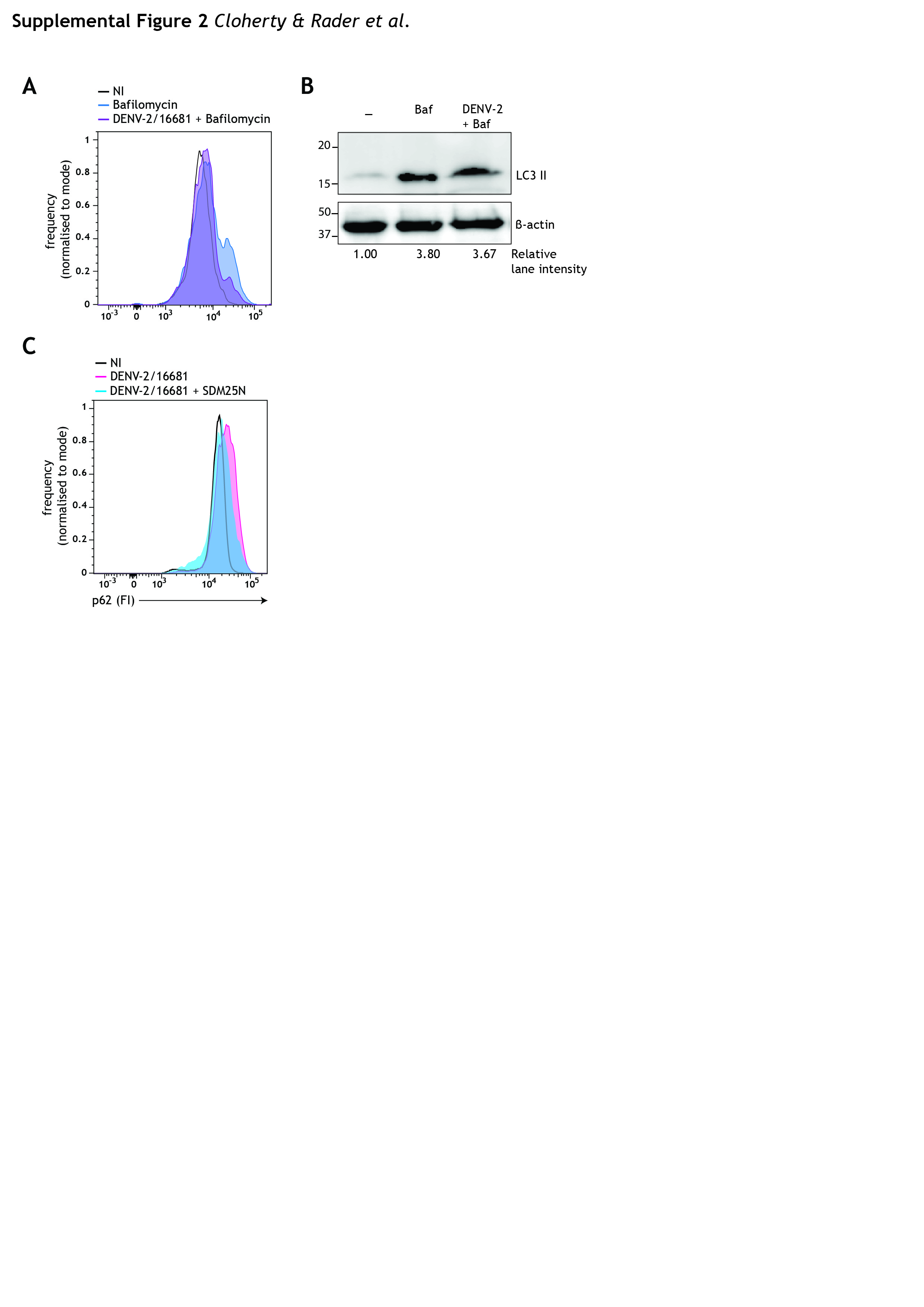

Supplement: Supplementary Figure 2 — Lack of autophagy flux in DENV-infected human DCs. (A, B) Autophagy activity in primary human DCs either left untreated, treated with bafilomycin A1, or concomitantly treated with bafilomycin A1 (50nM) and infected with DENV-2/16681 for 48 h, determined by intracellular LC3-II levels using saponin extraction (26, 27, 52, 58) (A), or by immunoblotting for LC3 (B). Relative abundance of LC3II determined by normalizing to β-actin. (A, B) Data are representative of n = 3 donors. (C) Intracellular p62 levels in primary human DCs infected with DENV-2/16681 for 48 h, with or without concomitant treatment with flavivirus replication inhibitor SDM25N (10 µM). Data are representative flow cytometry plots of n = 2 donors, determined by intracellular p62 staining. [file Image_2.jpeg]

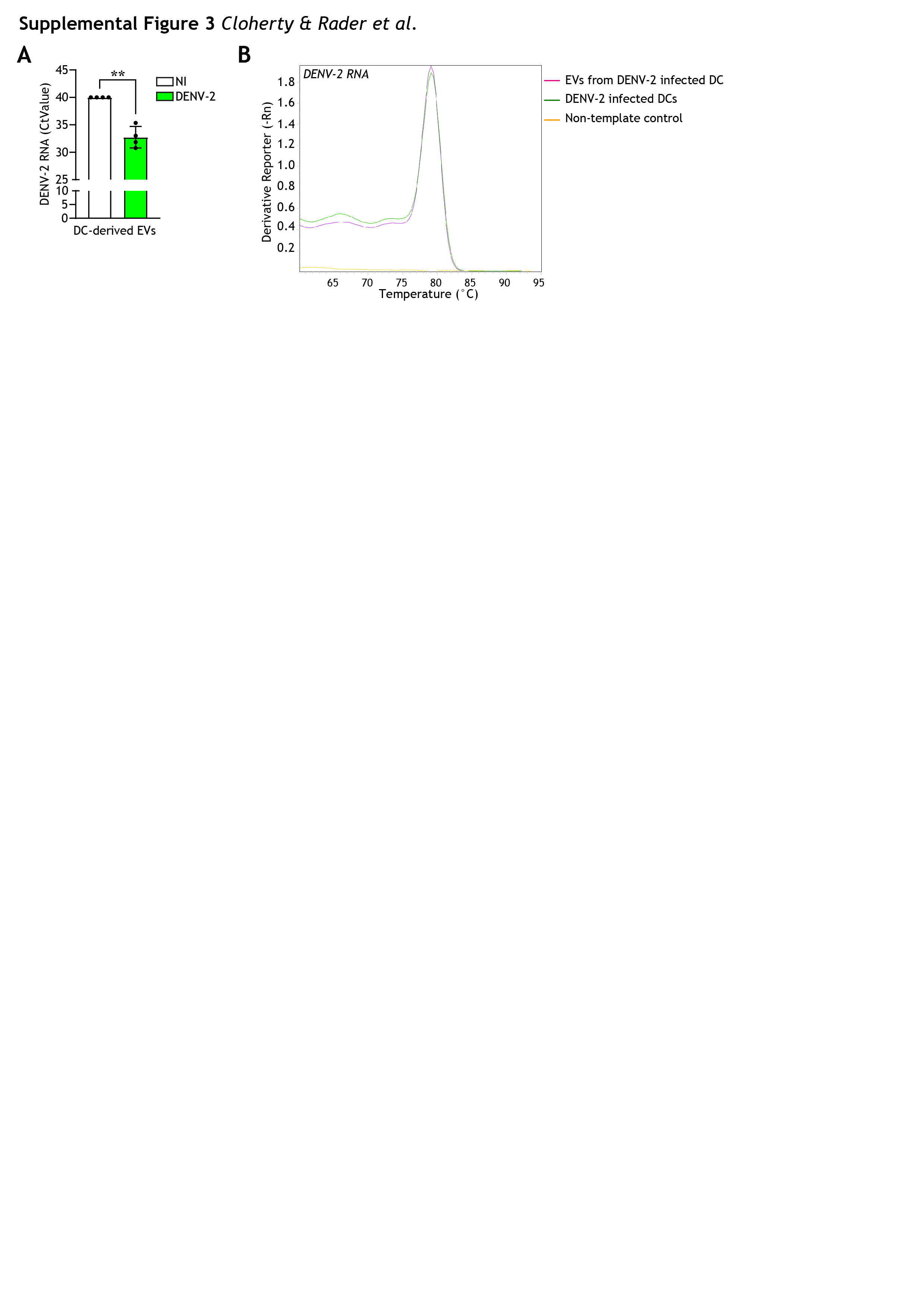

Supplement: Supplementary Figure 3 — Detection of LC3+ EVs released from the human U87.LC3-mCherry-GFP autophagy reporter cell line. (A) Graphical representation of the U87.LC3-mCherry-GFP cell line, which is typically utilised for monitoring of autophagy flux (26, 50, 70). LC3 is tandem tagged with both mCherry and GFP, resulting in dual fluorescence of intracellular autophagosomes in both red and green channels. Because GFP is more acid-sensitive than mCherry, a reduction in GFP signal is indicative of fusion of autophagosomes and lysosomes, i.e. autophagy flux. (B) Autophagy flux analyses in U87.LC3-mCherry-GFP cells, determined by whole-cell fluorescence measurement using imaging flow cytometry. U87.LC3-mCherry-GFP cells containing primarily autophagosomes that follow the canonical late stages of autophagy and fuse with lysosomes appear red due to the quenching of acid-sensitive GFP upon autophagosome-lysosome fusion, while the more acid-stable mCherry conjugated to LC3 remains bright. U87.LC3-mCherry-GFP cells containing primarily accumulated autophagosomes that do not follow the late degradative stages of autophagy express both green (GFP) and red (mcherry) fluorescence (26, 50, 70). (C) Use of the U87.LC3-mCherry-GFP autophagy reporter cell line for detection of extracellular LC3+ vesicles. Following overnight culture of U87.LC3-mCherry-GFP cells in FCS-depleted culture medium, conditioned medium (CM) was collected and pre-cleared by serial centrifugation (10 minutes at 2000x g followed by 30 minutes at 5000x g). EVs were immunomagnetically isolated using a pan-extracellular vesicle positive selection kit, and thereafter stained and analysed by imaging flow cytometry (62). General membrane labelling was performed using carboxyfluorescein succinimidyl ester (CFSE; 300 µM), and thereafter EVs were washed twice more to remove residual dye and antibody. To reduce swarming or coincident event detection during individual EV analysis, EV samples were serially diluted in PBS to determine an operational [file Image_3.jpeg]

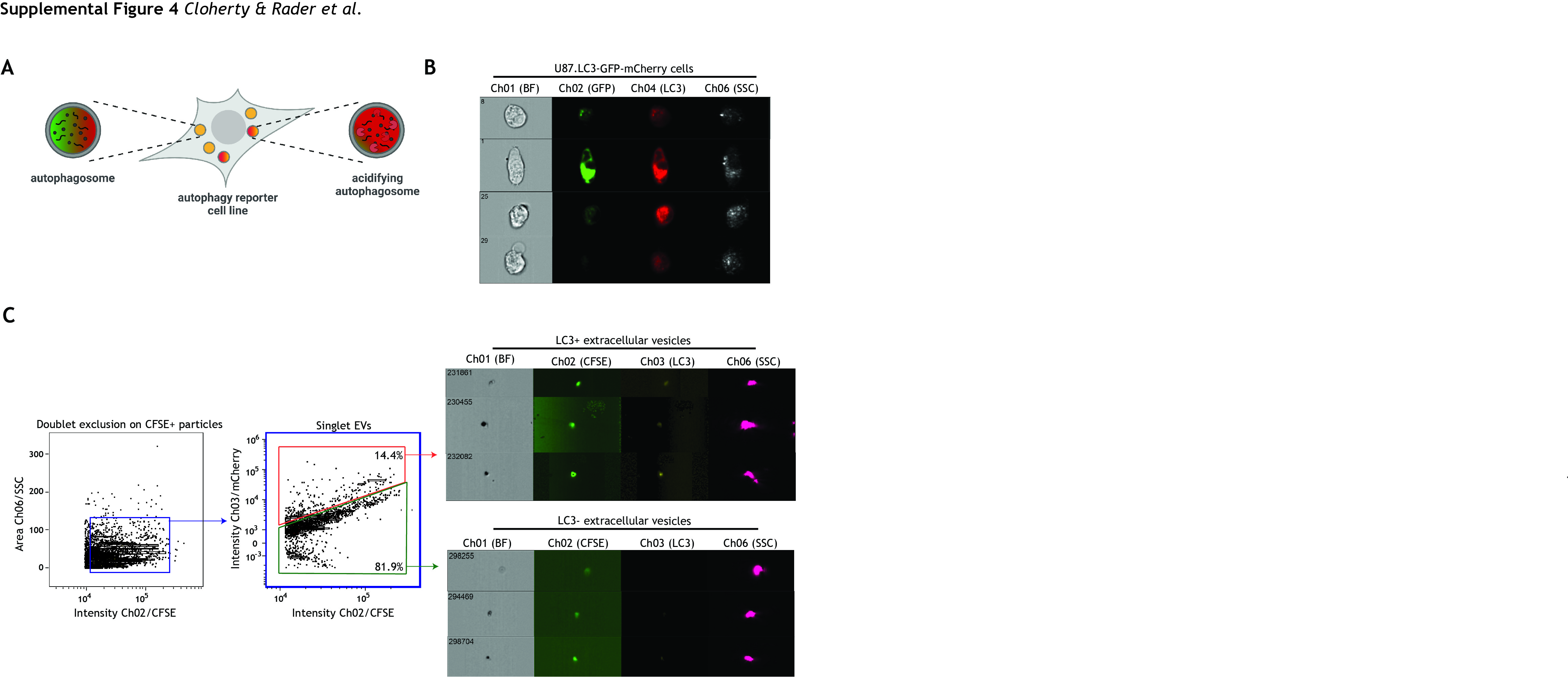

Supplement: Supplementary Figure 4 — DC-derived EVs harbour DENV-2 RNA. (A, B) Detection of DENV-2 RNA in DC-derived EVs, determined by quantitative real-time PCR. DCs were infected with DENV-2/16681 for 48 h. DCs were then extensively washed to remove input virus, and replated for 30 h. CM was then harvested, centrifuged to remove remaining DCs and cellular debris, and thereafter subjected to positive immunomagnetic selection of CD9/CD81/CD63+EVs. (A) Cycle threshold (Ct) values of viral RNA in isolated CD9/CD81/CD63+ EVs derived from either DENV-infected DCs or non-infected DCs. (B) Representative profiles of the melting curves of viral RNA amplicon in DENV-infected DCs (green) or isolated CD9/CD81/CD63+ EVs from DENV-infected DCs (fuchsia) or non-template amplification control (orange). (A, B) Data represents n=4 donors, **P < 0.01, student’s t-test. [file Image_4.jpeg]
